# First Insight into the Genome Sequence of Clostridium vincentii DSM 10228, Isolated from Sediment of the McMurdo Ice Shelf, Antarctica

**DOI:** 10.1128/genomeA.00334-18

**Published:** 2018-04-12

**Authors:** Anja Poehlein, Simon Bolz, Berenice Fischer, Rolf Daniel

**Affiliations:** a Genomic and Applied Microbiology & Göttingen Genomics Laboratory, Institute of Microbiology and Genetics, Georg-August University of Göttingen, Göttingen, Germany; b Applied Bioinformatics in Microbiology Course of the Microbiology and Biochemistry MSc/PhD Program, Georg-August University of Göttingen, Göttingen, Germany

## Abstract

Clostridium vincentii is an obligate anaerobic, saccharophilic, psychrophilic, Gram-positive, motile, and rod-shaped bacterium. It was isolated from a pond sediment of the McMurdo Ice Shelf, Antarctica. C. vincentii produces acetate and formate as main fermentation products. The draft genome consists of one chromosome (3.506 Mb) with 3,379 predicted protein-encoding genes.

## GENOME ANNOUNCEMENT

Clostridium vincentii DSM 10228 is an obligate anaerobe which was isolated from the sediment of a low-salinity pond located under cyanobacterial mats on the McMurdo Ice Shelf, Antarctica (1). This Gram-positive psychrophilic organism is capable of growing at temperatures ranging from 2 to 20°C, with optimal growth at 12°C (1). C. vincentii ferments saccharides and forms various products, such as acetate, formate, and butyrate (1).

For the isolation of C. vincentii DSM 10228 chromosomal DNA, the MasterPure purification kit was used as recommended by the manufacturer (Epicentre, Madison, WI, USA). Subsequently, the extracted DNA was used to generate Illumina paired-end sequencing libraries. Sequencing was performed using a MiSeq instrument and the MiSeq reagent kit version 3 according to the protocols of the manufacturer (Illumina, San Diego, CA, USA). Quality trimming was conducted using Trimmomatic version 0.36 (2), resulting in 2,064,492 paired-end reads. *De novo* genome sequence assembly was performed with SPAdes version 3.11.1 (3), which yielded 90 contigs (>500 b) with an average coverage of 175-fold. The results were validated with Qualimap version 2.2.1 (4).

The draft genome of C. vincentii consists of one chromosome (3.506 Mb) exhibiting an overall G+C content of 30.7%. Automatic gene prediction and identification of rRNA and tRNA genes were performed using the software tool Prokka (5), resulting in 11 rRNA genes, 78 tRNA genes, and 1 repeat region. In addition, 860 hypothetical genes and 2,519 protein-encoding genes with a predicted function were identified. Twelve of these genes were associated with prophages, 19 genes with multidrug resistance, and 4 genes with clustered regularly interspaced short palindromic repeat (CRISPR)/Cas systems.

C. vincentii is able to metabolize diverse substrates, including the monosaccharides fructose, glucose, galactose, *N*-acetylglucosamine, and xylose, the disaccharides lactose, maltose, mannose, and sucrose, and the polysaccharide xylan (1). In the draft genome sequence, putative genes coding for different phosphotransferase systems (PTS) and genes encoding the enzymes mannose-6-phosphate isomerase and alpha-xylosidase were present. Fermentation of the above-mentioned carbohydrates results in the products acetate, formate, and butyrate. Accordingly, potential genes encoding butyrate kinase, acetate kinase, acetyltransferase, and formate acetyltransferase were present.

### Accession number(s).

This whole-genome shotgun project has been deposited at DDBJ/EMBL/GenBank under the accession number PVXQ00000000. The version described here is version PVXQ01000000.
